# Multi-Species Transcriptomics of the Mammalian Stomach Reveals Conserved Programs and Ruminant Chamber Specialization

**DOI:** 10.3390/biology15191677

**Published:** 2026-09-22

**Authors:** Qi-Xuan Huang, Zhi-Hui Wang, Wen-Tian Wei, Si-Rui Liu, Yi-Lin Bai, Yue-Yu Bai, Meng-Hua Li, Song-Song Xu

**Affiliations:** 1 Frontiers Science Center for Molecular Design Breeding (MOE), State Key Laboratory of Animal Biotech Breeding, College of Animal Science and Technology, China Agricultural University, Beijing 100193, China; b20213040311@cau.edu.cn (Q.-X.H.); zhihui.wang@cau.edu.cn (Z.-H.W.); weiwt@cau.edu.cn (W.-T.W.); cauliusirui@163.com (S.-R.L.); menghua.li@cau.edu.cn (M.-H.L.); 2 Key Laboratory of Innovative Utilization of Local Cattle and Sheep Germplasm Resources (Co-Construction by Ministry and Province), Ministry of Agriculture and Rural Affairs, Zhengzhou University, Zhengzhou 450001, China; bylin213@zzu.edu.cn (Y.-L.B.); baiyy666@henu.edu.cn (Y.-Y.B.); 3 Institute of Livestock Breeding and Reproduction, Henan University, Zhengzhou 450046, China

**Keywords:** mammalian stomach, comparative transcriptomics, RNA-seq, ruminants, cross-species

## Abstract

The mammalian stomach has conserved digestive functions but also shows major anatomical and physiological differences among species. However, broad comparative transcriptomic analyses across mammals and across ruminant stomach chambers remain limited. Here, we collected and integrated 111 bulk RNA-seq datasets from 11 mammalian species (humans, mice, monkeys, dogs, horses, donkeys, pigs, cattle, deer, goats, and sheep) and analyzed 12,048 shared one-to-one orthologous genes. We identified 56 conserved genes mainly involved in translation, ribosome biogenesis, protein homeostasis, and mitochondrial function. Pairwise comparisons revealed extensive transcriptional differences, with 2070–7110 differentially expressed genes across species pairs, together with distinct species-associated transcription-factor activity patterns. We also identified 25 ruminant-enriched genes associated with epithelial differentiation, ion transport, mucosal defense, and metabolism. Analysis of 69 samples from the rumen, reticulum, omasum, and abomasum of sheep, goats, cattle, and deer showed clear chamber-associated transcriptomic differences. The abomasum had a distinct expression program, with 235 enriched genes related to hormone regulation, secretion, transport, and digestion. Weighted gene co-expression network analysis further identified different gene programs in the forestomach and abomasum. Together, these findings reveal conserved, species-associated, and chamber-associated transcriptional features of the mammalian stomach and provide a useful framework for understanding its molecular diversity.

## Simple Summary

This study compared gene expression patterns in stomach tissues from 11 mammalian species. We identified a group of genes that were consistently expressed across species and mainly involved in basic cellular functions. In contrast, many genes showed different expression patterns among species, reflecting the diversity of mammalian stomach biology. Ruminants also showed a distinct set of genes related to epithelial function, ion transport, defense, and metabolism. We further compared the four stomach chambers of sheep, goats, cattle, and deer. The rumen, reticulum, and omasum constitute the forestomach and showed relatively similar gene-expression patterns, whereas the abomasum, the glandular true stomach, was clearly distinct and was mainly associated with secretion and digestion. These findings indicate that the mammalian stomach shares conserved molecular programs while also exhibiting species- and chamber-associated differences, providing useful information for understanding mammalian digestive diversity and ruminant stomach specialization.

## 1. Introduction

The stomach is an important organ for digestion, nutrient absorption, epithelial barrier function, and immune defense. Stomach structure and function differ among mammalian species because of differences in diet and digestive physiology. These differences may be reflected in gene expression patterns.

Ruminants have a specialized four-chamber stomach comprising the rumen, reticulum, omasum, and abomasum. The rumen, reticulum, and omasum constitute the forestomach, whereas the abomasum is the glandular true stomach [1,2]. This anatomical organization enables microbial fermentation of fibrous plant material before subsequent enzymatic digestion in the abomasum [2]. Specifically, the rumen and reticulum work in coordination to drive microbial fermentation, volatile fatty acid absorption, particle sorting, and reticuloruminal motility [1,2,3]. In contrast, the omasum and abomasum perform distinct functional roles: the omasum mainly contributes to the absorption of water, electrolytes, and volatile fatty acids as well as the regulation of digesta flow, whereas the abomasum is responsible for gastric acid secretion and enzymatic and proteolytic digestion [1,2,3].

Recent advances in genomics and multi-omics have provided important resources for understanding genetic diversity and functional traits in ruminant livestock [4,5]. Previous comparative studies have shown that mammalian organs contain both conserved gene-expression programs and species-associated expression patterns. Conserved genes usually support basic cellular functions, whereas species-associated genes may contribute to differences in organ structure and physiology [6,7,8,9]. However, most previous studies focused on a limited number of species or on a single ruminant species. A broad comparison of stomach transcriptomes across mammals is still lacking. In addition, the transcriptomic differences among the four stomach chambers across different ruminant species remain unclear.

To address these questions, we collected 111 publicly available stomach RNA-seq datasets from 11 mammalian species, including humans, mice, monkeys, dogs, horses, donkeys, pigs, cattle, deer, goats, and sheep. We mapped genes from different species to shared one-to-one orthologous genes and constructed a comparative mammalian stomach transcriptome atlas. We then analyzed global transcriptomic relationships, pairwise differential gene expression, conserved genes, species-associated genes, and transcription-factor activity patterns.

We further focused on sheep, goat, deer, and cattle to compare the four stomach chambers. We identified chamber-associated genes, performed GO enrichment analysis, and used WGCNA to identify co-expression modules related to forestomach and abomasum functions. This study provides a comparative framework for understanding the conserved and specialized transcriptional programs of mammalian and ruminant stomach tissues.

## 2. Materials and Methods

### 2.1. Data Collection

Publicly available bulk RNA-seq data from mammalian stomach tissues were obtained for 11 species, yielding 111 samples in total. The dataset included goats [10], sheep [10], horses [10], donkeys [10], deers [10], cattle [10,11], mice [12], pigs [13], humans [14], monkeys [15], and dogs [15]. For ruminant species, samples from the rumen, reticulum, omasum, and abomasum were included, whereas stomach samples, including specific gastric regions when annotated, were used for monogastric species. Sample information included species, breed, age or developmental stage, sex, sequencing platform, accession number, and data source. Detailed information for all samples is provided in Table S1.

### 2.2. Quality Control and Read Processing

Raw sequencing reads in FASTQ format were first examined with FastQC v0.12.1 to evaluate overall sequencing quality. Reads containing adapters or low-quality sequences were filtered using fastp v0.23.2 [16] with default settings. The filtered reads were then mapped to the reference genome of each species with STAR v2.7.10b [17]. Uniquely aligned reads were used for gene-level quantification with featureCounts in Subread v2.0.3 [18], together with the corresponding GTF annotation files. The resulting count matrices were used as the input for subsequent transcriptomic analyses.

### 2.3. Cross-Species Ortholog Mapping

For cross-species comparisons, orthologous genes were retrieved from Ensembl (http://dec2021.archive.ensembl.org/index.html; Dec 2021 v105, (accessed on 31 July 2026)) using the R package biomaRt v2.57.1 (https://github.com/Huber-group-EMBL/biomaRt, (accessed on 3 August 2026)). The resulting orthologous genes were concatenated and integrated based on human proteins, yielding a total of 12,048 orthologous genes across the 11 species. The final dataset included humans, mice, monkeys, dogs, horses, donkeys, pigs, cattle, deer, goats, and sheep. Genes without a known human orthologue in the Ensembl or NCBI database were retained in the count matrix without conversion.

### 2.4. Expression Matrix Preprocessing and Normalization

Following ortholog mapping, the expression matrices were integrated based on the shared one-to-one orthologous genes. Raw expression matrices were normalized using the variance-stabilizing transformation (VST) implemented in DESeq2 v1.46.0 [19]. The VST-transformed expression matrix was used for t-SNE, Pearson correlation analysis, conserved gene analysis, and stomach chamber-associated transcriptomic analyses.

### 2.5. Differential Expression and GO Enrichment Analysis

Only genes meeting the minimum expression criteria (≥10 read counts and detected in at least three samples) were retained for differential expression analysis. Pairwise comparisons among the 11 mammalian species were performed using DESeq2 v1.46.0 [19] based on the 12,048 shared one-to-one orthologous genes. Multiple-testing correction was performed using the Benjamini–Hochberg method. Genes with an FDR < 0.05 and absolute log_2_ fold change > 1 were defined as differentially expressed genes (DEGs). GO enrichment analysis of the DEGs was conducted using clusterProfiler v4.14.6 [20]. GO terms with an adjusted *p* value < 0.05 were considered significantly enriched.

### 2.6. Conserved Gene Analysis

To identify genes with conserved expression patterns across mammalian stomach tissues, Tau, coefficient of variation (CV), and mean expression level were calculated from the species-level mean expression matrix. Tau and CV were used to identify genes with broad and stable expression across species, whereas the mean expression filter was used to reduce the influence of low-expression noise. Genes with Tau < 0.1, CV < 0.1, and mean expression > 5 were defined as conserved genes. The mean expression > 5 threshold was chosen to exclude genes with very low abundance, because such genes can appear to have low CV but provide less stable biological interpretation. This analysis identified 56 conserved genes. GO enrichment was assessed using clusterProfiler v4.14.6 [20], and the conserved gene expression-correlation network was constructed using Pearson correlation analysis.

### 2.7. Transcription Factor Activity Analysis

Transcription factor (TF) activity was estimated using decoupleR v2.17.0 [21] with TF–target interactions from the DoRothEA database in the dorothea package v1.23.0 [22]. For cross-species comparison, the 12,048 shared orthologous genes were represented by human gene symbols, and their mean expression levels were calculated for each species. The resulting species-level expression matrix was analyzed using the univariate linear model (ULM) implemented in decoupleR [21,23]. The regulatory direction of each TF–target interaction was used as the weight, and the t-value obtained from the model was used as the TF activity score. Only DoRothEA interactions with confidence levels A–C were included, and TF regulons with fewer than five represented target genes were removed. TF activity scores were then standardized across species. For visualization, the six TFs with the highest positive standardized scores in each species were selected and displayed in a heatmap.

### 2.8. Species-Associated and Ruminant-Enriched Gene Analysis

Genes detected exclusively in one species were identified from the union expression matrix. Species-enhanced homologous genes were identified from the shared homologous gene matrix according to species-associated expression differences. For the ruminant comparison, deer, goat, sheep, and cattle were compared with the seven non-ruminant species. Genes with higher expression in all four ruminant species and low expression in all non-ruminant species were defined as ruminant-enriched genes. Ruminant-enriched genes were selected using an adjusted *p* value < 0.05, a log2 fold change > 1, a mean expression level ≥ 1 in each ruminant species, and a mean expression level ≤ 1 in each non-ruminant species.

### 2.9. Comparative Transcriptomic Analysis of the Four Ruminant Stomach Chambers

To investigate stomach chamber specialization, RNA-seq samples from sheep, goat, deer, and cattle were classified into four stomach chambers: rumen, reticulum, omasum, and abomasum. Rumen, reticulum, and omasum were collectively defined as the forestomach. Principal component analysis was performed using the transformed expression matrix to evaluate chamber-associated transcriptomic patterns. Chamber-associated expression differences were assessed using a linear model including stomach chamber class and species as explanatory variables. Abomasum-enriched genes were selected using an adjusted *p* value < 0.05, a log2 fold change > 1, a mean expression level ≥ 1 in the abomasum, and a maximum mean expression level ≤ 1 in the forestomach. This analysis identified 235 abomasum-enriched genes. GO enrichment analysis was performed using the genes tested in the chamber-associated model as the background gene set.

### 2.10. Weighted Gene Co-Expression Network Analysis (WGCNA)

Weighted gene co-expression network analysis (WGCNA) was performed using the WGCNA R package v1.74 [24] to examine co-expression patterns among the four ruminant stomach chambers. Gene-expression values were log2-transformed, and biological replicates were averaged for the rumen, reticulum, omasum, and abomasum of sheep, goats, deer, and cattle. Sample and gene quality were checked using the goodSamplesGenes function. A weighted co-expression network was then built with the blockwiseModules function using an unsigned network. The soft-thresholding power was set to 20, with a minimum module size of 300 genes and deepSplit = 2. Similar modules were merged using a module eigengene dissimilarity threshold of 0.25. Module eigengenes were calculated and correlated with stomach chamber groups using Pearson correlation analysis, and corresponding *p* values were obtained using corPvalueStudent. Module membership was calculated for each gene based on its correlation with the corresponding module eigengene. Genes with high module membership were considered candidate hub genes.

### 2.11. Statistical Analysis and Visualization

All analyses were performed using R 4.6.0. t-SNE analysis was performed using the Rtsne v0.17 package. Heatmaps were generated using ComplexHeatmap v2.22.0 and related visualization packages. Other plots were generated using ggplot2 v3.5.1. Pearson correlation coefficients were calculated for sample correlation and module trait analyses. *p* values were adjusted using the Benjamini–Hochberg method where applicable.

## 3. Results

### 3.1. Comparative Structure of Mammalian Stomach Transcriptomes

We collected 111 publicly available stomach RNA-seq datasets, representing 11 mammalian species (humans, mice, monkeys, dogs, horses, donkeys, pigs, cattle, deer, goats, and sheep) (Figure 1A). The resulting union matrix contained 194,271 genes, and the shared homologous matrix contained 12,048 genes. The species-level sample numbers were 28 for sheep, 14 for goats, 14 for deer, 13 for cattle, 12 for pigs, 8 for humans, 6 for mice, 6 for monkeys, 6 for dogs, 2 for donkeys, and 2 for horses. Detailed sample metadata, including age, sex, sequencing platform, and corresponding references, are provided in Table S1.

To enable cross-species comparative analyses, genes from each species were mapped to their corresponding human orthologs using Ensembl BioMart (Ensembl release 105) through the R package biomaRt v2.57.1). In total, 12,048 orthologous genes were shared among all 11 mammalian species (Figure 1B). Among the human-centered comparisons, mouse shared the largest number of genes with humans (19,022 genes), whereas dogs shared the fewest (14,913 genes) (Table S2).

Following ortholog mapping, the gene expression matrices from 11 mammalian species were integrated based on 12,048 shared orthologous genes. To reduce the influence of sequencing depth and library-size differences, raw count matrices were normalized using the variance-stabilizing transformation (VST) implemented in DESeq2. The resulting VST-transformed expression matrix was used for downstream analyses, including t-distributed stochastic neighbor embedding (t-SNE) and Pearson correlation analysis.

As shown in Figure 1C, replicates from the same species generally clustered closely together in the t-SNE space, whereas samples from different species occupied distinct regions, indicating strong within-species reproducibility and substantial transcriptomic divergence among the 11 mammalian species. The clear species-associated separation suggests that the integrated transcriptomic dataset retained robust biological signals after normalization by the variance-stabilizing transformation implemented in DESeq2. Pearson correlation analysis based on the VST-transformed expression matrix further showed consistently higher correlation coefficients among replicates within the same species than among samples from different species (Figure 1D). Notably, sheep and goat samples displayed higher transcriptomic similarity than any other interspecies comparison (Figure 1D). This pattern is consistent with their close phylogenetic relationship and shared ancestry as closely related ruminant species. Collectively, these results demonstrate the reproducibility and consistency of the integrated stomach RNA-seq dataset and support its suitability for subsequent comparative analyses of conserved, species-associated, and species-specific gene-expression programs.

### 3.2. Differential Gene Expression Across Mammalian Stomach Transcriptomes

Following ortholog mapping, pairwise differential expression analyses were performed for all 55 species pairs using the 12,048 shared orthologous genes. All 55 comparisons produced substantial numbers of DEGs, ranging from 2070 to 7110 genes (Figure 2).

As shown in Figure 2A, significant expression differences were broadly distributed across all pairwise comparisons. Both positive and negative log_2_ fold changes were detected, indicating that transcriptional divergence involved bidirectional changes in gene expression. Several comparisons showed large numbers of genes with substantial expression changes, demonstrating extensive divergence in the expression levels of homologous genes despite their conservation across species.

The number of upregulated and downregulated DEGs for each comparison is summarized in Figure 2B. The smallest number of DEGs was identified between cattle and goat, including 959 upregulated and 1111 downregulated genes. Other relatively low DEG counts were observed for cattle versus deer, cattle versus sheep, deer versus goat, and goat versus sheep, with 2130, 2164, 2220, and 2393 DEGs, respectively. In contrast, mouse versus donkey showed the largest number of DEGs, including 1085 upregulated and 6025 downregulated genes, for a total of 7110 DEGs. Other comparisons involving donkey also showed extensive divergence, including sheep versus donkey with 6314 DEGs, goat versus donkey with 6217 DEGs, dog versus donkey with 6176 DEGs, and cattle versus donkey with 5902 DEGs.

Overall, the numbers of upregulated and downregulated genes were broadly comparable across the 55 comparisons, indicating that the observed differences were not dominated by a unidirectional change in expression. Together, Figure 2A,B demonstrate that the presence of 12,048 shared homologous genes does not imply conservation of their expression levels. Instead, the mammalian stomach transcriptome is characterized by widespread species-associated transcriptional divergence, with relatively smaller differences among several ruminant species and particularly pronounced divergence in comparisons involving donkey.

The pattern is compatible with the idea that evolutionary diversification of organ physiology can arise primarily through changes in gene regulation rather than the gain or loss of large numbers of core genes [7,8,25].

### 3.3. Identification of Evolutionarily Conserved Genes in Mammalian Stomach Tissues

We next evaluated the expression characteristics of the 12,048 shared homologous genes using three complementary metrics: Tau, coefficient of variation (CV), and mean expression level. The Tau distribution was mainly concentrated in the low-to-moderate range, indicating that many homologous genes showed relatively broad expression across mammalian stomach tissues (Figure 3A). Most genes exhibited relatively low CV values, suggesting limited interspecies variation in their expression levels (Figure 3B). Mean expression levels were mainly distributed in the low-to-moderate range, with a smaller group of highly expressed genes retained using the threshold of mean expression > 5 (Figure 3C).

Genes with Tau < 0.1, CV < 0.1, and mean expression > 5 were considered highly conserved. The intersection of these three criteria identified 56 conserved genes (Figure 3D and Table S3). These genes showed broad expression, low interspecies variability, and relatively high expression levels across mammalian stomach tissues.

The conserved gene set was mainly composed of genes involved in fundamental cellular maintenance processes. Translation and ribosome associated genes included *EEF2*, *EIF3A*, *EIF4A3*, *EIF4B*, *EIF4G1*, *PABPC1*, *LARP1*, and multiple ribosomal protein genes, including *RPL3*, *RPL4*, *RPL5*, *RPL7A*, *RPL10A*, *RPL19*, *RPLP0*, *RPS4X*, *RPS6*, *RPS12*, *RPS14*, *RPS18*, *RPS27A*, and *RPSA*. Genes involved in protein folding, degradation, and quality control included *CCT8*, *HSPA9*, *PSMB4*, *PSMB5*, *PSMD8*, *UBQLN1*, *UBA1*, *RNF5*, *DERL1*, and *TXNDC5*. Several conserved genes were associated with mitochondrial structure and energy metabolism, including *HADHA*, *MDH1*, *SUCLG1*, *NDUFA8*, *IMMT*, *TMEM70*, *CHCHD2*, *MRPL20*, and *MRPL28*. In addition, *AP3D1*, *ARCN1*, *ARF4*, *ERGIC3*, *SCAMP2*, *RABGGTB*, *GDI2*, and *TSG101* are involved in intracellular vesicle trafficking, endoplasmic reticulum-Golgi transport, or membrane protein sorting. Genes such as *RHOA*, *CMTM6*, *KRTCAP2*, and *PTMA* are related to cytoskeletal organization, membrane-associated signaling, and cellular structural maintenance.

GO enrichment analysis showed significant enrichment for cytoplasmic translation and ribosome biogenesis (Figure 3E and Table S4). Additional enriched terms were related to regulation of translation, translational initiation, rRNA processing, and cell-fusion- and myoblast-fusion-related processes. Because these cell-fusion-related terms were mainly driven by overlapping ribosomal protein genes, they should be interpreted as annotation-associated functional signals rather than evidence of a stomach-specific myoblast-fusion program.

To further characterize co-expression relationships among the conserved genes, an expression-correlation network was constructed using the conserved gene set (Figure 3F). All 56 conserved genes were considered as candidate nodes. The displayed network retained the 90 strongest expression-correlation edges, connecting 41 of the 56 conserved genes; the remaining 15 genes had no retained edges and were therefore not displayed. The network was dominated by ribosomal proteins and translation-associated factors, including *RPL4*, *RPL5*, *RPL10A*, *RPL19*, *RPLP0*, *RPS6*, *RPSA*, *EEF2*, *EIF3A*, *EIF4A3*, *EIF4G1*, and *PABPC1*. Other connected genes were associated with protein degradation, chaperone-mediated protein folding, mitochondrial function, and intracellular trafficking.

Collectively, these findings demonstrate that mammalian stomach tissues possess a conserved transcriptional core characterized by stable expression of genes involved in translation, ribosome biogenesis, protein folding and degradation, mitochondrial metabolism, intracellular trafficking, and cytoskeletal maintenance. These genes likely support the basic cellular and metabolic requirements shared across mammalian stomach tissues rather than representing chamber-specific functional genes.

### 3.4. Species-Specific and Species-Enhanced Transcriptional Signatures

To examine species-associated expression patterns, we classified genes into two groups: genes detected exclusively in one species and shared homologous genes showing enhanced expression in a particular species. The number of exclusively detected genes varied substantially, ranging from 155 in dog to 32,610 in human, likely reflecting differences in genome annotation, gene-model completeness, and species-specific transcript representation (Figure 4A). Species-enhanced homologous genes were fewer but were identified in all 11 species, ranging from 64 in sheep to 1654 in donkey.

To identify genes enriched in ruminant species, we compared the expression of deer, goat, sheep, and cattle with those of the seven non-ruminant species. Genes showing consistently higher expression across the four ruminant species but low or undetectable expression in the non-ruminant species were selected as ruminant-enriched genes. This strategy identified 25 genes, which were visualized in Figure 4B. The selected genes included *SPINK9*, *SDK2*, *SDS*, *FGF5*, *HAND1*, *CCL22*, *PAPOLB*, *UROC1*, *TGM5*, *EDAR*, *SERTM1*, *TMPRSS11D*, *PAX9*, *SLC26A3*, *BNC1*, *WNT3A*, *SLC6A4*, *CA1*, *SLC4A9*, *PGLYRP4*, *FAT2*, *FOXE1*, *SHBG*, *DIPK1C*, and *PADI1* (Table S5). The 25 genes showed consistently higher expression across deer, goat, sheep, and cattle than across the seven non-ruminant species (Figure 4B). These genes were mainly associated with epithelial differentiation, ion transport, mucosal defense, and metabolic processes.

Representative species-associated functional signatures were further highlighted in Figure 4C. Mouse-associated genes were enriched in digestion, regulation of hormone levels, and digestive-system processes, suggesting a transcriptional program related to digestive and endocrine functions. Monkey-associated genes were enriched in mononuclear cell differentiation, B-cell activation, and lymphocyte differentiation, indicating an immune-related expression signature. Horse-associated genes were mainly related to plasma-membrane transport, sodium-ion transport, and responses to external stimuli, suggesting differences in epithelial ion transport and environmental sensing. Pig-associated genes were enriched in responses to biotic stimuli, responses to lipopolysaccharide, and steroid hormone secretion, reflecting potential differences in mucosal immune and endocrine regulation. Goat-associated genes were enriched in epidermis development, skin development, and sulfur-compound metabolic processes, suggesting differences in the keratinized foregut epithelium and sulfur-related metabolic functions. Together, these representative enrichments indicate that species-associated stomach expression patterns involve digestive, immune, epithelial transport, endocrine, and metabolic processes. Because these functional categories were derived from heterogeneous public datasets, they should be interpreted as species-associated transcriptional signatures rather than definitive species-specific stomach functions.

We therefore inferred transcription-factor (TF) activity in each species using the DoRothEA resource and the decoupleR package and identified species-associated TF activity patterns (Figure 4D). The mouse group was enriched for TFs associated with metabolic regulation, epithelial function, and ion homeostasis, including PPARA, SREBF1, HNF1A, and VDR. The human group was enriched for TFs associated with innate immune responses, cellular differentiation, and chromatin regulation, including IRF3, KLF9, and THAP1. The monkey group was enriched for TFs related to immune regulation, transcriptional activation, and cell-cycle progression, including BATF, E2F1, and ETS1. The dog group was enriched for TFs associated with tissue differentiation and transcriptional regulation, including ETV1, HOXB13, and FOXJ2. The horse group was enriched for TFs related to endodermal specification, gastrointestinal epithelial development, and tissue patterning, including GATA4, FOXA2, and GLI2. The donkey group was enriched for TFs associated with circadian regulation and muscle differentiation, including CLOCK and MYOD1. The pig group was enriched for TFs related to lipid metabolism and epithelial transcriptional regulation, including PPARG, POU2F1, and USF1. The deer group was enriched for TFs associated with epithelial differentiation, DNA-damage responses, vascular regulation, and stress-related transcription, including TP63, TP53, ERG, and JUNB. The goat group was enriched for TFs related to steroid-responsive transcription, epithelial differentiation, and tissue development, including AR, SOX9, and ERG. The sheep group was enriched for TFs associated with developmental regulation, tissue differentiation, and chromatin-related processes, including FOXL2, NFIC, and ZEB2. The cattle group was enriched for TFs related to hormone-responsive transcription, TGF-β signaling, and cell-cycle regulation, including ESR1, SMAD3, and E2F4. These results demonstrate that mammalian stomach tissues exhibit distinct species-associated transcriptional regulatory programs in addition to a conserved core transcriptome.

### 3.5. Transcriptomic Specialization of the Four Ruminant Stomach Chambers

We next analyzed the four stomach chambers of sheep, goat, cattle, and deer using 69 samples, including 40 rumen, 10 reticulum, 9 omasum, and 10 abomasum samples. PCA showed clear chamber-associated separation while retaining species-related expression patterns (Figure 5A). Abomasum samples formed the most distinct transcriptional group, whereas rumen, reticulum, and omasum samples showed greater proximity, consistent with the functional distinction between the forestomach and the abomasum [1].

The first heatmap displayed 25 ruminant-enriched genes identified by comparing sheep, goat, deer, and cattle with seven non-ruminant species (Figure 5B). These genes showed consistently higher expression in ruminants but relatively low or undetectable expression in non-ruminants. Across the four stomach chambers, most of these genes showed higher expression in the rumen, reticulum, and omasum than in the abomasum, indicating that the expression of ruminant-enriched genes was further influenced by stomach-chamber identity.

The second heatmap showed all 235 abomasum-enriched genes. These genes exhibited relatively low expression in the rumen, reticulum, and omasum but higher expression in the abomasum across the four ruminant species (Figure 5B and Table S6). Among these genes, several have previously been reported to be involved in gastric, glandular, epithelial, or secretory functions and were highlighted on the right side of the heatmap. GO enrichment analysis of the 235 abomasum-enriched genes revealed significant enrichment for regulation of hormone levels, hormone transport, peptide hormone secretion, peptide secretion, hormone secretion, peptide transport, signal release, protein secretion, protein localization to extracellular region, and digestion (Figure 5C and Table S7). These results indicate that the abomasum-enriched gene set is mainly involved in secretory activity, peptide and hormone processing, extracellular protein transport, and digestive functions.

WGCNA identified distinct co-expression patterns among the 16 species–chamber combinations (Figure 5D). For biological interpretation, rumen, reticulum, and omasum were considered forestomach compartments. The module–chamber correlation patterns showed that forestomach and abomasum tissues were associated with different co-expression programs, although the strength of these associations varied among species. In particular, the M7 module showed relatively stronger associations with forestomach compartments in goat and deer and negative associations with abomasum tissues, whereas the M5 module showed its strongest positive association with cattle abomasum (r = 0.91, *p* < 0.001).

GO enrichment analysis further revealed different functional characteristics of M7 and M5 (Figure 5E). M7 was enriched for cytoplasmic translation, translation, protein biosynthetic process, ribonucleoprotein complex biogenesis, cellular respiration, respiratory electron transport chain, and mitochondrial ATP synthesis coupled electron transport. These terms indicate a coordinated program involving protein synthesis, ribosome-related processes, and mitochondrial energy production, consistent with the metabolic and epithelial demands of forestomach tissues [26,27]. In contrast, M5 was enriched for digestion, digestive system process, protein O-linked glycosylation, glycoprotein biosynthetic process, glycoprotein metabolic process, and organic anion transport. These functions are consistent with a glandular and secretory epithelial program involving digestive activity, protein modification, and membrane transport.

Collectively, the 25-gene set represents a transcriptional signature shared by ruminant species, whereas the 235-gene set represents an abomasum-enriched expression program. The WGCNA results further demonstrated that forestomach- and abomasum-associated tissues were characterized by distinct co-expression modules and functional programs. Together, these findings indicate that ruminant stomach specialization reflects both species-group-associated gene expression and chamber-specific transcriptional regulation.

## 4. Discussion

In this study, we built a comparative transcriptomic atlas of the mammalian stomach. The atlas included 111 RNA-seq datasets from 11 mammalian species. We identified 12,048 shared one-to-one orthologous genes. Samples from the same species clustered closely in the t-SNE analysis and showed high Pearson correlation values, whereas samples from different species were clearly separated. These results indicate distinct gene expression patterns among species and high consistency among samples within the same species. Sheep and goat showed the highest transcriptomic similarity, which is consistent with their close phylogenetic relationship and similar ruminant physiology [28,29].

All 55 comparisons between species showed many differentially expressed genes. The smallest number of DEGs was found between cattle and goat, whereas the largest number was found between mouse and donkey. Several comparisons among ruminants showed relatively fewer DEGs. These results show that shared homologous genes do not have the same expression levels in all species. Instead, changes in gene-expression regulation may contribute to differences in stomach structure and function during mammalian evolution [7,8,25].

We identified 56 highly conserved genes shared by all 11 species. These genes were mainly associated with core cellular maintenance processes, including translation and ribosome biogenesis, protein quality control, mitochondrial function, intracellular trafficking, and cytoskeletal maintenance. The GO enrichment results supported these functional patterns. The gene association network mainly contained genes related to ribosomes and translation. Some genes were also related to protein processing and mitochondrial function. Therefore, these conserved genes mainly support basic cellular functions in the mammalian stomach. We also identified species-associated expression patterns. The number of genes detected in only one species differed substantially among species. We further identified 25 ruminant-enriched genes, which showed higher expression in deer, goat, sheep, and cattle than in the non-ruminant species. These genes were mainly related to epithelial differentiation, ion transport, mucosal defense, and metabolism. Several of these genes have been reported to be associated with rumen epithelial function. *CA1*, *SLC26A3*, and *SLC4A9* are associated with ion transport, anion handling, and short-chain fatty acid uptake in the rumen epithelium [30]. TGM5 is expressed in differentiating keratinocytes and contributes to the formation of the cornified cell envelope, supporting its role in epithelial differentiation and barrier formation [31]. *PGLYRP4* has also been reported to show preferential expression in the sheep rumen and may contribute to epithelial innate defense [32]. The functions of these reported genes are consistent with the epithelial, transport, and defense requirements of the ruminant forestomach. However, the functions of the remaining genes in ruminant stomach tissues require further investigation.

The TF analysis identified different regulatory patterns among species. Metabolism-related TFs included PPARA, PPARG, and SREBF1, which are involved in lipid and energy metabolism [33,34]. Epithelial differentiation was represented by TP63, a key regulator of epithelial development and differentiation [35]. Immune-related TFs included IRF3, which is involved in innate antiviral responses [36]. Endodermal development was associated with FOXA2 and GATA4, which regulate endoderm specification and foregut development [37]. Cell cycle regulation was represented by E2F1 and E2F4, which regulate cell proliferation and cell cycle progression [38]. Stress-related regulation included TP53 and JUNB, which are involved in DNA damage responses, cellular stress, inflammation, and differentiation [39,40].

The four stomach chambers of sheep, goat, deer, and cattle showed clear differences in their transcriptomic profiles. In the PCA, abomasum samples were clearly separated from rumen, reticulum, and omasum samples, whereas the three forestomach chambers showed greater similarity. This pattern agrees with the different physiological roles of the forestomach and the abomasum [29].

The 235 abomasum-enriched genes were mainly associated with hormone regulation, peptide secretion, protein secretion, peptide transport, and digestion, which are consistent with the secretory and digestive roles of the abomasum. *HNF4A* is involved in gastric epithelial differentiation and tissue homeostasis [41]. *GAST* encodes gastrin, a hormone produced by gastric G cells that plays an important role in gastric acid secretion and mucosal growth and differentiation [42]. *PGA5* encodes pepsinogen A5, which is produced and secreted by gastric chief cells and contributes to protein digestion following its conversion to pepsin [43].

WGCNA further identified different module patterns among the stomach chambers. Module 7 was positively associated with the forestomach samples of goat and deer and was enriched in translation, ribonucleoprotein complex biogenesis, cellular respiration, respiratory electron transport chain, and mitochondrial ATP synthesis coupled electron transport. Module 5 showed the strongest positive association with cattle abomasum and was enriched in digestion, protein O-linked glycosylation, glycoprotein biosynthetic process, glycoprotein metabolic process, and organic anion transport. These results show different functional patterns between the goat and deer forestomach and the cattle abomasum.

Several limitations should be acknowledged. First, this study used publicly available bulk RNA-seq datasets, and some observed expression differences may partly reflect variation in cell composition among samples. Second, sample sizes were uneven across species, particularly for donkey and horse, and results from these low-sample groups should therefore be interpreted with appropriate caution and warrant further validation in larger sample sets. Third, independent qRT-PCR validation was not performed because matched stomach tissues from the corresponding species and stomach chambers were not available, and the key genes identified here require further experimental validation. Fourth, although species-specific genes were retained in species-level analyses, only one-to-one orthologs were used for cross-species comparisons, which limits the direct evaluation of lineage-specific genes and gene families potentially relevant to ruminant physiology. Finally, differences among public datasets in breed, age, sex, developmental stage, sampling site, and sequencing protocol may also influence gene expression patterns. Future studies using standardized samples and single-cell or spatial transcriptomic data are needed to validate our findings.

## 5. Conclusions

In this study, we built a comparative transcriptomic atlas of the mammalian stomach using 111 RNA-seq datasets from 11 species. The shared homologous genes showed both conserved and species-associated expression patterns. The 56 conserved genes mainly supported basic cellular functions, including translation, protein processing, mitochondrial activity, and intracellular transport. We also identified 25 genes with higher expression in ruminants than in non-ruminants. These genes were mainly related to epithelial function, ion transport, mucosal defense, and metabolism. The four stomach chambers of sheep, goat, deer, and cattle also showed clear transcriptomic differences. The rumen, reticulum, and omasum had similar expression patterns, whereas the abomasum showed a distinct program related to secretion and digestion. Overall, our results provide a comparative transcriptomic basis for understanding the conserved functions, species-associated differences, and chamber specialization of the mammalian stomach.

## Figures and Tables

**Figure 1:**
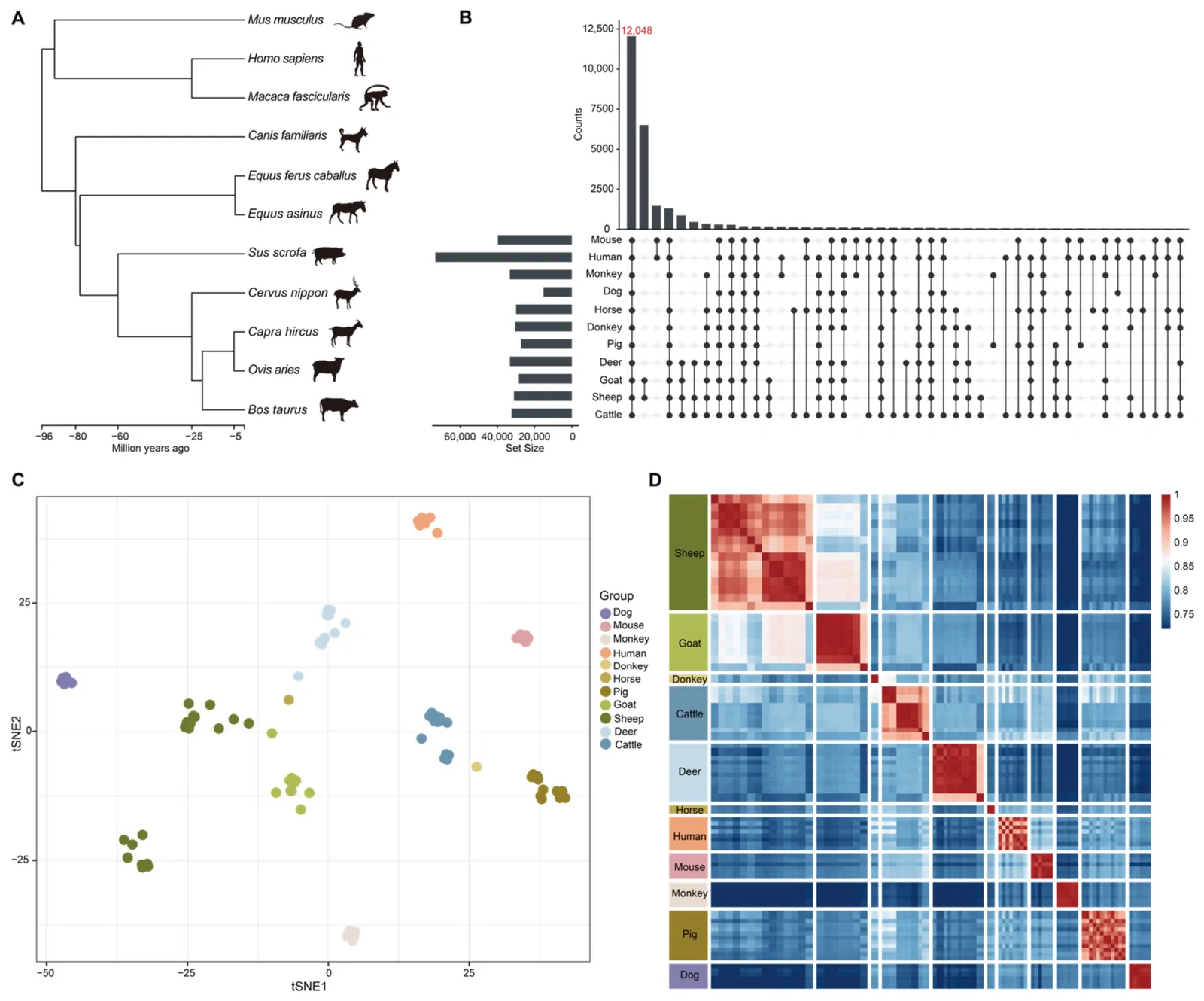
Overview of the comparative mammalian stomach transcriptome atlas. (**A**) Phylogenetic tree of 11 mammalian species. The divergence times (million years ago, MYA) are indicated at the nodes, with the root representing approximately 96 MYA. Branch lengths are proportional to evolutionary distance. (**B**) UpSet plot showing the numbers of one-to-one orthologous genes identified in 11 species. (**C**) t-distributed stochastic neighbor embedding (t-SNE) clustering of 111 RNA-seq samples. (**D**) Pearson correlation heatmap of 111 samples across 11 mammalian species.

**Figure 2:**
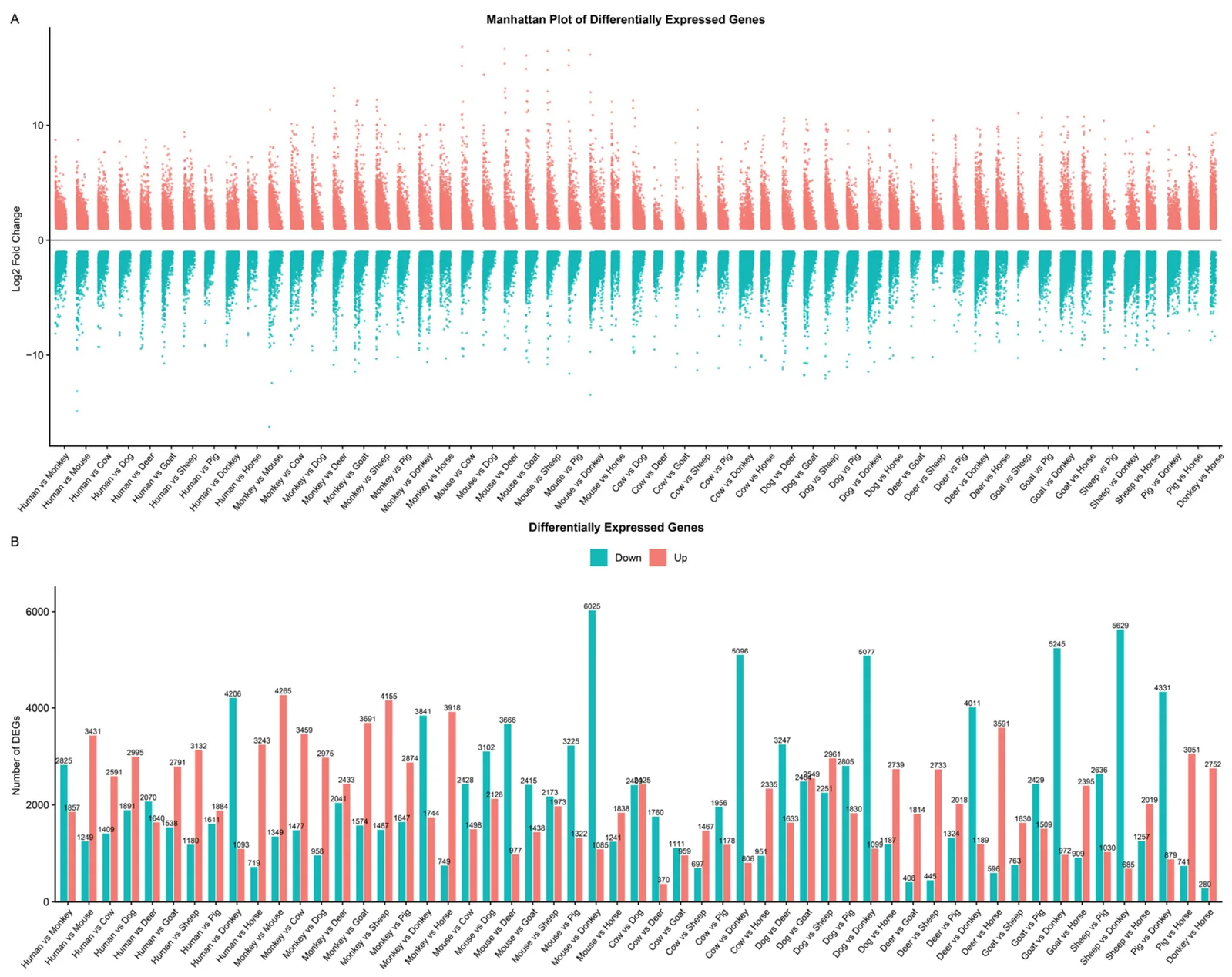
Differential gene expression across 11 mammalian species. (**A**) Overview of upregulated and downregulated DEGs across all pairwise comparisons. (**B**) Counts of DEGs identified from pairwise species comparisons.

**Figure 3:**
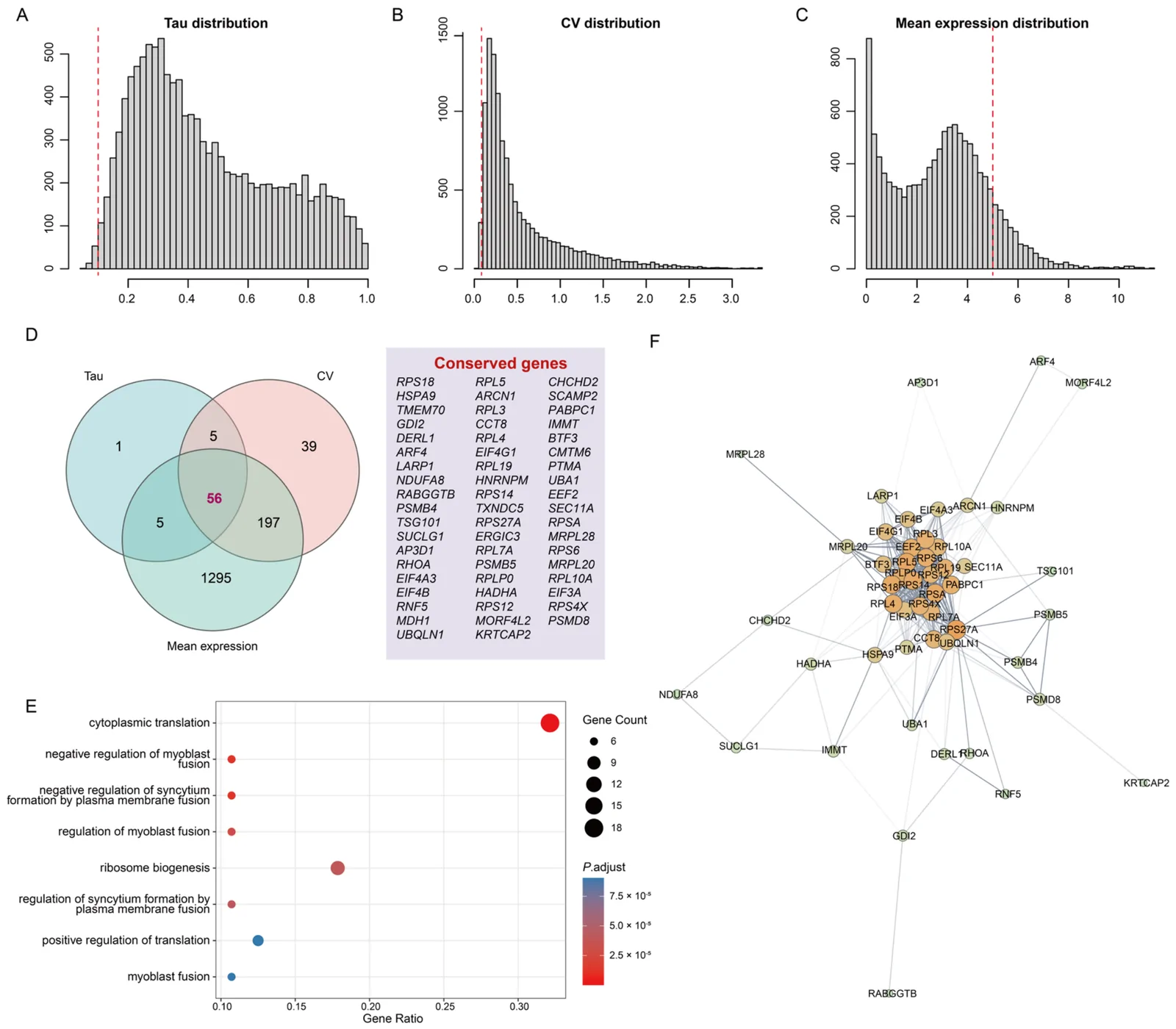
Conserved expression programs across mammalian stomach. (**A**) Histogram of distribution of Tau values for 12,048 one-to-one orthologous genes; the dashed line indicates the Tau = 0.1 threshold. (**B**) Histogram of distribution of coefficient of variation (CV) for 12,048 one-to-one orthologous genes; the dashed line indicates the CV = 0.1 threshold. (**C**) Histogram of distribution of mean expression for 12,048 one-to-one orthologous genes; the dashed line indicates the mean expression = 5 threshold used to exclude low-expression genes. (**D**) Venn diagram showing genes satisfying Tau < 0.1, CV < 0.1, and mean expression >5, yielding 56 conserved genes. (**E**) Gene Ontology biological-process enrichment of the conserved genes. (**F**) Gene expression-correlation network of the conserved genes.

**Figure 4:**
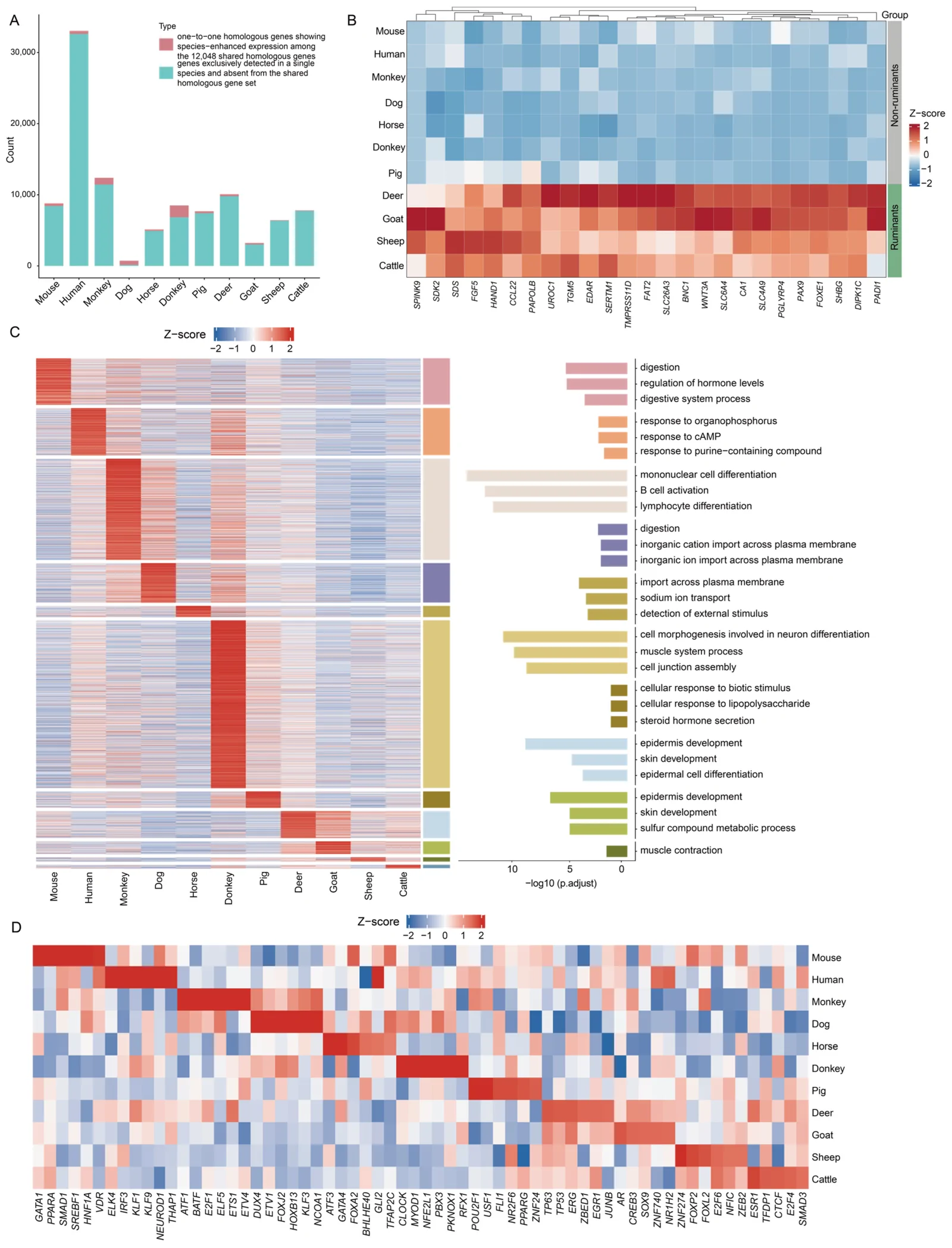
Identification of species-specific gene expression signatures. (**A**) Species-specific and species-enhanced genes across 11 mammalian species. Green bars represent species-specific genes (expressed only in the given species), while red bars represent species-enhanced genes (expressed across all species but with elevated expression specifically in the indicated species). (**B**) Expression heatmap of the 25 ruminant-enriched genes across 11 mammalian species. (**C**) Expression heatmap and representative GO biological-process enrichment of species-associated genes across 11 mammalian species. (**D**) Heatmap showing species-associated TF activity patterns, displaying the six TFs with the highest activity scores for each species.

**Figure 5:**
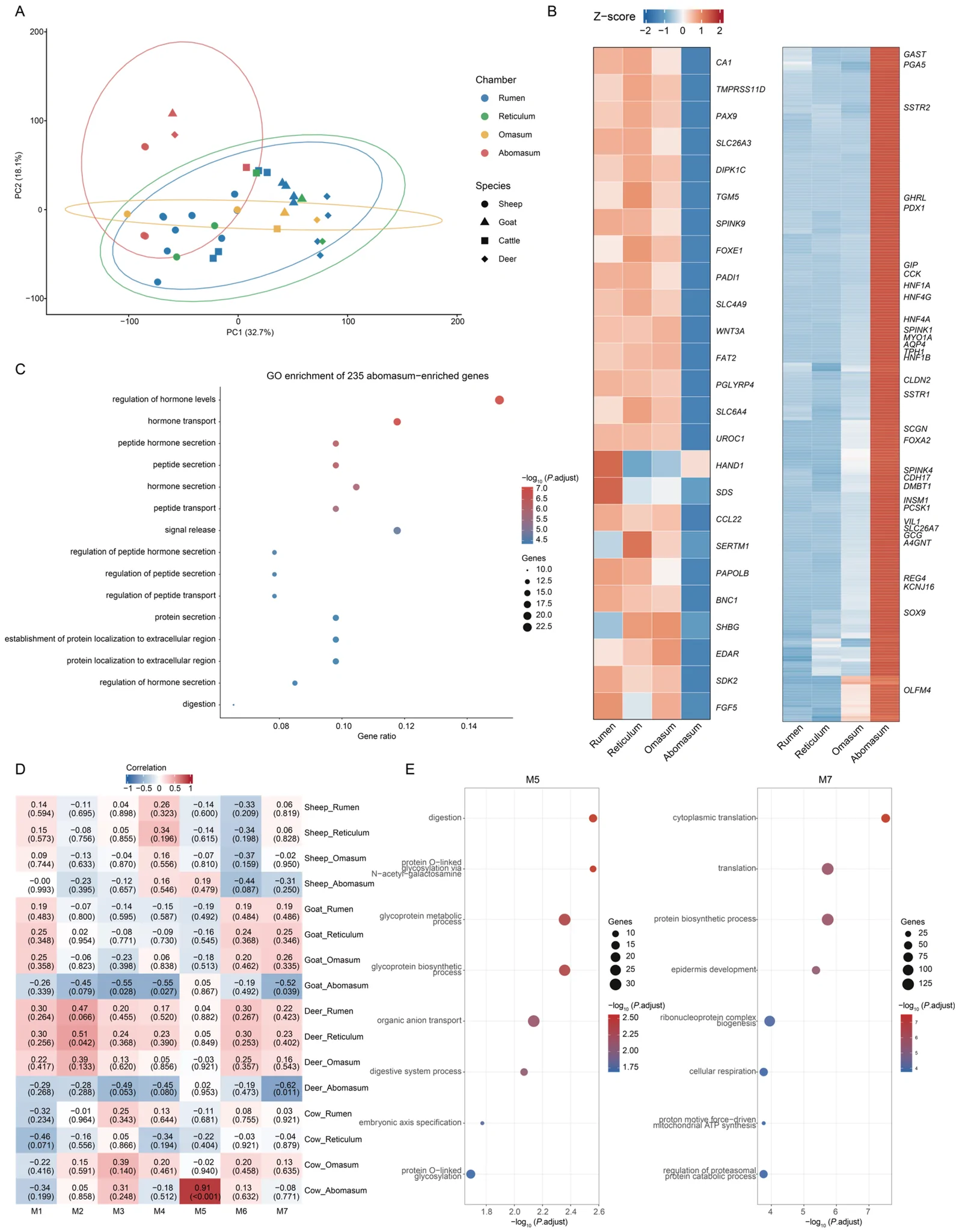
Comparative transcriptomics of the four ruminant stomach chambers. (**A**) PCA of 69 samples from sheep, goat, cattle, and deer across rumen, reticulum, omasum, and abomasum. (**B**) Heatmaps showing the expression patterns of 25 ruminant-enriched genes (left) and 235 abomasum-enriched genes (right) across the four stomach chambers. Gene labels on the right indicate abomasum-enriched genes with previously reported gastric, glandular, epithelial, or secretory functions. (**C**) GO biological process enrichment analysis of the 235 abomasum-enriched genes. (**D**) WGCNA heatmap of gene co-expression networks across the 16 species–chamber combinations. (**E**) GO biological process enrichment analysis of genes in the M5 and M7 co-expression modules.

## Data Availability

The RNA-seq expression matrices and sample metadata used in this study were obtained from the public datasets listed in Supplementary Table S1.

## Supplementary Materials

The following supporting information can be downloaded at: https://www.mdpi.com/article/10.3390/biology15191677/s1, Table S1: Metadata and publication sources of the 111 gastric RNA-seq samples from 11 mammalian species used in this study; Table S2: Homologs of human genes found in the genomes of the other ten species; Table S3: Conserved_genes (Tau < 0.1, CV < 0.1, and mean expression >5); Table S4: Gene Ontology biological-process enrichment of the conserved genes; Table S5: 25 genes showing high expression in all four ruminant species but low or undetectable expression in non-ruminants; Table S6: In the four ruminant species, 235 genes exhibited relatively low expression levels in the rumen, reticulum, and omasum, but higher levels in the abomasum; Table S7: Gene Ontology biological-process enrichment of the 235 abomasum-enriched genes.
